# Correlation of radiomorphometric indices of the mandible and mandibular angle fractures

**DOI:** 10.1016/j.heliyon.2022.e10549

**Published:** 2022-09-07

**Authors:** Aida Karagah, Reza Tabrizi, Fatemeh Pourahmadali, Ahad Alizadeh, Maryam Tofangchiha, Romeo Patini

**Affiliations:** a Department of Oral and Maxillofacial Surgery, Qazvin University of Medical Sciences, Qazvin, Iran; b Department of Oral and Maxillofacial Surgery, Shahid Beheshti University of Medical Sciences, Tehran, Iran; c Student Research Committee, Qazvin University of Medical Sciences, Qazvin, Iran; d Medical Microbiology Research Center, Qazvin University of Medical Sciences, Qazvin, Iran; e Department of Oral and Maxillofacial Radiology, Qazvin University of Medical Sciences, Qazvin, Iran; f Department of Head, Neck and Sense Organs, School of Dentistry, Catholic University of Sacred Heart, Rome, Italy

**Keywords:** Mandibular fractures, Computed tomography, Mandibular angle, Radiomorphometric indices

## Abstract

This study assessed the correlation of radiomorphometric indices of the mandible and mandibular angle fractures (MAFs) in an Iranian population. This retrospective study was conducted on 3D computed tomography (CT) scans of 118 patients between 18 to 60 years. The images were divided into two groups with MAFs and other types of mandibular fractures (non-MAF). The gonial angle, ramus height, condylar neck width, minimum ramus width, and mandibular length were all measured using MARCO PACS software. Age, gender, and presence and eruption status of third molar at the fracture side were all recorded. The correlation between these parameters and MAF was analyzed using R software (alpha = 0.05). Of all patients, 41 samples had MAF. The two groups were not significantly different regarding the mean age and gender (P > 0.05). The mean size of gonial angle and ramus height in the MAF group were significantly larger, and smaller than the corresponding values in the non-MAF group, respectively (P < 0.001). The median minimum ramus width in the MAF group was significantly smaller than that in the non-MAF group (P = 0.001). Patients with a large gonial angle had 6.6 times higher odds of MAF compared with other fracture types (P = 0.046). Condylar neck width, mandibular length, and erupted third molars had no significant correlation with type of fracture. Presence of impacted third molar increased the odds of MAF by 5.55 times.

Patients with a large gonial angle, short ramus height, minimum ramus width, and impacted third molar are more susceptible to MAF. Surgeons can use these indices to predict the risk of MAF in trauma patients with such facial characteristics, and make a diagnosis by radiographic modalities.

## 1 Introduction

The human mandible has an important complex role in facial esthetics, speech, mastication, and deglutition [1, 2, 3]. Mandibular fractures are the second most common fractures in the maxillofacial region after nasal fractures, accounting for 19%–40% of all fractures in this region. They more commonly occur in the third decade of life in males [4, 5, 6, 7]. Such a high prevalence rate is due to the unique anatomy and characteristics of the mandible, especially its mobility and limited bone support compared with other facial bones as well as its prominent position [2,8,9].

The prevalence of mandibular fractures is increasing due to motor vehicle accidents, occupational accidents, falls, sport injuries, and violence [10]. High rate of mandibular fractures could be explained by the unique characteristics of the mandible such as its mobility and limited bone support, compared with other facial bones [11]. A number of factors are implicated in the occurrence of mandibular fractures such as direction and magnitude of load, biomechanical properties such as bone density and normal or pathological anatomical structure of bone creating weak areas, loads applied by the muscles of mastication, patterns of occlusal loads, and normal anatomical variations that cause weak points [2,9,12]. The pattern of mandibular fracture also depends on the point of impact and surface area of impact [12].

Mandibular angle fracture (MAF) refers to a type of fracture posterior to the second molar tooth that occurs between the body of mandible and the ascending ramus, where impacted or semi-impacted mandibular third molars are often present [9,13]. MAFs comprise 12%–30% of all mandibular fractures [8]. MAFs most commonly occur in 26–40-year-old individuals (69.7%) followed by 18–25-year-olds (30.3%) [14]. Such a high incidence rate is due to the presence of curvature at this site, presence of impacted third molars, mandibular height, thinner cross-sectional area, low bone density, and changed orientation of the trabecular pattern of bone in this region [5,15,16].

The skeletal properties of the face are unique. Nonetheless, some key classifications are used to categorize these properties [1]. Different skeletal measurements are available for facial growth patterns [7]. The gonial angle (GA) is an anthropometric parameter used for assessment of the mandibular growth pattern [8]. Low gonial angle (LGA), normal gonial angle (NGA), and high gonial angle (HGA) correspond to horizontal, normal, and vertical growth, respectively [17]. Also, evidence shows that a vertical growth pattern is correlated with a shorter ramus height [7]. A positive correlation exists between the GA and the bony structure of the mandibular angle [5]. The cross-sectional thickness of the muscles of mastication in HGA individuals is lower than that in LGA, and NGA individuals. Also, mandibular height, masticatory force, and alveolar cortical thickness are inversely correlated with the size of GA [17].

The posterior position and biomechanics of the mandibular angle complicate the treatment of MAFs. That is why MAFs cause greater complications compared with other mandibular fracture types. The prevalence of these complications can reach 32% of population [18]. Management of MAFs is difficult due to complex mechanics of the mandibular angle such as thin cross-section, abrupt change in the path of curvature, presence of third molars, and attachment of the muscles of mastication applying loads along different vectors [19].

A number of studies have reported a positive association between third molar impaction and MAF [12,20,21]. However, studies on the correlation of morphometric indices of the mandible and MAF are limited. This study aimed to assess the correlation of radiomorphometric indices of the mandible with MAFs in order to find out whether the risk of MAF can be predicted according to radiomorphometric indices of the mandible.

## 2 Materials and methods

This study was approved by ethical committee of Qazvin University of Medical Sciences with ethical number of IR. QUMS.REC.1400.214 and there is no conflict with ethical considerations.

This retrospective study was conducted on 3D computed tomography (CT) scans of patients retrieved from the archives of the Oral and Maxillofacial Radiology Department of Qazvin Shahid Rajaie Hospital. The patients had presented to this center due to mandibular fracture between 2016 and 2021.

The sample size in each group was calculated according to the results of a previous pilot study. In the pilot study, 10 specimens were allocated to each group. The GA was considered as the main primary predictor in this study. Accordingly, the total sample size was calculated to be 34 assuming alpha = 0.05, and study power of 0.8.

The inclusion criteria were patients with mandibular fractures whose preoperative CT scans were available, and aged between 18 and 60 years.

The exclusion criteria were CT scans with artifacts compromising accurate measurements, edentulous patients, patients with no posterior occlusal support, syndromic subjects and/or subjects with facial malformations, patients with osteoporosis using bisphosphonates or other antiresorptive medications, and bilateral involvement or displacement of fracture segments; all these cases were excluded as they do not allow accurate measurements.

In this study, morphometric indices served as the predictor variable, and MAF was the outcome variable. Presence of a fracture line behind the second molar and along the curvature connecting the body of mandible to the posterior border of ramus was considered as a MAF in this study [22]. The following parameters were measured on 3D CT scans in the sagittal plane: the size of GA, ramus height, condylar neck width, minimum ramus width, mandibular length, third molar eruption status, and age and gender of patients. According to Bhullar *et al.* [23], and Laversha *et al.* [24], no significant difference was found in the size of GA between the right and left sides. Thus, measurements were made only at one side (preferably the intact side). The site of mandibular fracture was categorized into the following seven categories: angle, body, symphysis-parasymphysis, ramus, condyle, coronoid, and alveolar fractures [25]. To further simplify the statistical analyses and according to study objectives, the fractures were categorized into two main groups of MAF and non-MAF. All 3D CT scans had been taken with Somatom Emotion 16-slice 3D CT scanner with the exposure settings of 110 kV, 35 mAs, 8.18 s scan time, and 16 × 0.6 mm fine detector collimation in supine position. All images were observed on a 19-inch monitor (Samsung, Seoul, Korea).

All measurements were made on 3D CT scans stored in DICOM Standard format using MACRO PACS (Division Medical Application, Tahavolat Novin Yadman, Iran). The anatomical landmarks for the measurements were first identified by an oral and maxillofacial surgeon and an oral and maxillofacial radiologist. If the two observers did not agree on the location of the anatomical landmarks, an experienced oral and maxillofacial radiologist would guide them to reach a single decision. Next, the parameters were measured digitally by a trained senior dental student. The diagnosis of impaction of third molars was made by an oral and maxillofacial surgeon.

**Figure 1:**
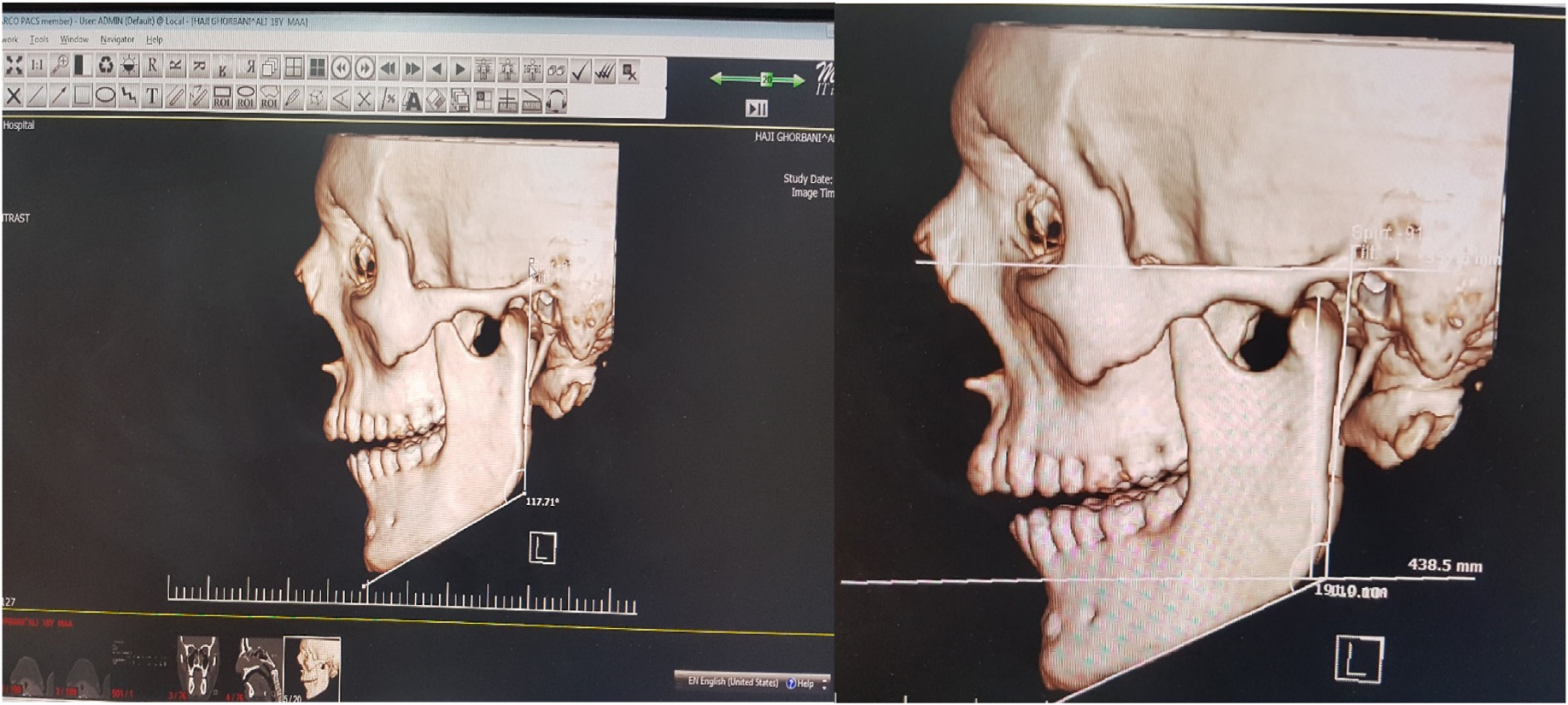
Measurement of GA (left side) and ramus height (right side) using MACRO PACS software.

Measurement of GA: The GA was considered as the angle formed between the line tangent to the posterior border of mandible and the line tangent to the inferior border of mandible (Figure 1A) [26]. The GA was categorized into the following three groups:

HGA: GA size >125°
NGA: 120 ≤ GA size ≤125°
LGA: GA size <120°

### 2.1 Ramus height

The distance between the highest point of the mandibular condyle and the ramus horizontal reference plane (which is a plane parallel to the Frankfurt horizontal plane that passes from Gonion, point of intersection of a line tangent to the posterior border of ramus and inferior border of mandible) (Figure 1B) [27,28].

### 2.2 Width of condylar neck

The shortest distance between the most concave point of the lateral border of the condylar neck and the most concave point of the internal border of the condylar neck [27].

### 2.3 Minimum ramus width

The minimum distance between the most concave point of the anterior border of ramus and the most concave point of the posterior border of ramus [27].

### 2.4 Mandibular length

The distance between the condylion (Co) and gonathion (Gn) [29]. Co is the most superior point on the condylar head [30], and Gn is the most anterior-inferior point of the chin [30].

### 2.5 Third molar status

Third molar status was categorized as missing, erupted (the crown was not covered with bone), or impacted (the crown was partially or completely covered with bone) [31].

Age and gender of patients were also recorded.

### 2.6 Statistical analysis

The data were analyzed by R software version 4.0.4. The morphometric indices of the mandible were compared between the two groups using t-test (for normally distributed data) and Mann-Whitney U test (for non-normally distributed data). The Chi-square test was applied to assess the correlation of gender, type of GA, and third molar impaction with type of mandibular fracture. Also, the logistic regression was used to estimate the odds ratios (OR) for each parameter. Level of significance was set at 0.05.

## 3 Results

All eligible 3D CT scans during the aforementioned period (n = 118) were evaluated. A total of 118 CT images of patients with mandibular fracture were evaluated; out of which, 41 were MAF and 77 were non-MAF. Accordingly, the prevalence of MAF in our study population was found to be 34.74%.

### 3.1 Demographics

Of all patients, 105 were males (88.98%) and 13 were females (11.02%), with a mean age of 27.25 ± 9.63 years. The mean age was 28.22 ± 10.62 years in the MAF and 26.73 ± 9.09 years in the non-MAF group, P = 0.448). The two fracture groups were not significantly different regarding age (t-test, P = 0.448) or gender (Chi-square test, P = 1.00).

### 3.2 Radiomorphometric indices of the mandible

Table 1 presents the radiomorphometric indices of the mandible in the two groups of fracture types. As shown, the mean GA in the MAF group was 8.81° larger than that in the non-MAF group (t-test, P < 0.001).

**Table 1:** Radiomorphometric indices of the mandible in the two groups of fracture types.

Variable	Total	MAF	Non-MAF	P-value
Gonial angle (degrees)∗	124.93 ± 7.32	130.68 ± 6.17	121.87 ± 5.92	<0.001
Ramus height (mm)∗	63.30 ± 7.27	57.36 ± 5.48	66.45 ± 6.04	<0.001
Condylar neck width (mm)†	11.14 ± 1.84	10.700 (9.800,11.900)	11.400 (10.300,12.400)	0.219
Minimum ramus width (mm)†	31.74 ± 4.23	30.400 (27.200,32.200)	33.100 (30.600,34.900)	0.001
Mandibular length (mm)∗	116.63 ± 13.32	115.22 ± 12.03	117.40 ± 13.97	0.379

∗ Mean ± SD of the variables was used and compared by t test.

† Median (IQR) of the variables was used and compared by Mann-Whitney U test.

Table 2 presents the frequency of different sizes of GA in the two fracture groups. As shown, the two groups were significantly different regarding the frequency of different GA sizes (P < 0.001), and the frequency of HGA was significantly greater in the MAF group. Based on Table 3, multiple analysis showed that in HGA patients, the odds of MAF were 6.6 times higher than those in non-MAF patients (OR = 6.6, 95% CI: [1.07, 41.66], P = 0.046). The OR of MAF in HGA patients was 20.520 times higher than that in NGA and LGA patients (P < 0.001, OR = 20.520, 95% CI: 7.073–59.532).

**Table 2:** Frequency of different sizes of GA in the two fracture groups.

Variable	Total	MAF	Non-MAF	P-value
HGA	56 (47.46%)	36 (87.8%)	20 (25.97%)	<0.001
LGA	25 (21.19%)	0 (0%)	25 (32.47%)	
NGA	37 (31.36%)	5 (12.2%)	32 (41.56%)	

The results are reported as count (%) and evaluated by Chi-squared test.

**Table 3:** Estimating odds ratio using multiple Logistic regression analysis.

Age		OR (95% CI)	P-value
		1.02 (0.95,1.1)	0.589
Gender	Female∗	1	-
	Male	0.37 (0.04,3.56)	0.397
Third molar	Missing∗	1	-
	Erupted	0.51 (0.1,2.55)	0.413
	Impacted	5.55 (1.08,29.52)	0.043
GA		1.11 (0.94,1.33)	0.214
Ramus height		0.58 (0.45,0.75)	<0.001
Condylar neck width		1.33 (0.79,2.21)	0.282
Minimum ramus width		0.99 (0.78,1.26)	0.959
Mandibular length		1.08 (0.97,1.21)	0.168
GA	NGA∗	1	-
	LGA	0.36 (0.01,9.83)	0.555
	HGA	6.6 (1.07,41.66)	0.046

∗ Female and Missing of third molar were used as a reference level for their own variables.

The mean ramus height was significantly lower in MAF than non-MAF group (P < 0.001). Multiple analysis showed that by each 1 mm increase in ramus height, the OR of MAF decreased by 42% compared with that in non-MAF group (P < 0.001, OR = 0.58, 95% CI: 0.45–0.75).

Also, the minimum ramus width in the MAF group was significantly smaller than that in the non-MAF group (Mann-Whitney U test, P = 0.001). No other significant differences were noted between the two fracture groups (P > 0.05).

### 3.3 Relationship of third molar impaction with fracture type

Table 4 presents the frequency distribution of third molar eruption status in the two fracture groups. The Chi-square test showed a significant difference in third molar status between the two groups (P = 0.008) such that the frequency of third molar impaction was higher in the MAF group. Regression analysis showed that impaction of third molar increased the odds of MAF by 5.5 times compared with non-MAF (P = 0.043, OR = 5.5, 95% CI: 1.08–29.52) (Table 3).

**Table 4:** Frequency distribution of third molar status in the two fracture groups.

Variable		Total	MAF	Non-MAF	P-value
Third molar status	Missing	31 (26.27%)	11 (26.83%)	20 (25.97%)	0.008
	Erupted	43 (36.44%)	8 (19.51%)	35 (45.45%)	
	Impacted	44 (37.29%)	22 (53.66%)	22 (28.57%)	

The results are reported as count (%) and evaluated by Chi-squared test.

## 4 Discussion

This study aimed to assess the correlation of radiomorphometric indices of the mandible with MAFs in order to find a way to predict the risk of MAF and its prevention in susceptible individuals. CT does not have the shortcomings of 2D radiography, and enables more accurate assessment of the details in the maxillofacial region [32].

The mean age of patients with mandibular fracture in this study was 27.25 ± 9.63 years, which was in line with other studies [2,3,15,33,34]. The two groups of MAF and non-MAF had no significant difference in the mean age, which was similar to the study by Dhara *et al.* [5], but different from the findings of Semel *et al.* [9]. ] concluded that patients with MAF were significantly younger than patients with other mandibular fracture types. Difference between their results and the present findings in this respect may be due to the different sample sizes.

In the present study, 88.98% of patients were males and 11.02% were females, which was in line with other studies [2,3,15,33,34]. Also, the MAF and non-MAF groups were not significantly different regarding gender, which was in line with the findings of Dhara *et al.* [5]. Such a high prevalence of fracture in males may be due to their more frequent encounter with risky situations of mandibular fracture.

The GA is an important anthropometric parameter which is involved in facial esthetics, and biodynamics of the masticatory muscles [15]. In the present study, the mean size of GA in MAF group was 8.81° larger than that in non-MAF group. Accordingly, a significant association was noted between larger size of GA and occurrence of MAF (P < 0.001). This result was in agreement with the findings of Panneerselvam *et al.* [15], Elias *et al.* [7], and Dhara *et al.* [5], and different from the results of Shroff *et al.* [2]. In the study by Panneerselvam *et al.* [15], 210 panoramic images of patients with mandibular fracture were evaluated, and the mean GA in the MAF group was 4.5° larger than that in the non-MAF group (P = 0.000). Elias *et al.* [7] evaluated 100 CT scans of patients with mandibular fracture, and reported that the GA in the MAF group was larger than that in the non-MAF group. Dhara *et al.* [5] assessed 70 panoramic radiographs of patients with mandibular fracture, and reported that the GA size was 10.2° larger in the MAF group (P = 0.000). Shroff *et al.* [2] evaluated 294 panoramic radiographs of patients with mandibular fracture, and reported that the mean GA in the MAF group was 0.9° larger than that in the non-MAF group, but this difference was not significant. Different findings of the latter study may be due to the fact that GA is not the only variable implicated in the occurrence of MAF, and other factors such as the severity of trauma, the intensity of impact, muscle traction, and presence of third molar should also be taken into account. GA is a valuable index for assessment of growth pattern and rotation of the mandible. Downward and backward rotation of the mandible results in HGA, while upward and forward rotation of the mandible results in LGA [35]. Osato *et al.* [36] defined LGA as GA < 120° and HGA as GA > 125° [].

The present study showed that the odds of MAF (compared with non-MAF) were 6.6 times higher in HGA patients. Also, the odds of MAF in HGA patients were 20.52 times the rate in NGA and LGA patients. These values were 11.7 and 8.7 in studies by Panneerselvam *et al.* [15], and Dhara *et al.* [5], respectively, and were statistically significant. However, this value was 0.502 in the study by Shroff *et al.* [2], and not statistically significant, which was different from the present findings. LGA, NGA, and HGA correspond to horizontal, normal, and vertical growth patterns, respectively. According to CT and ultrasound, the cross-section of the masticatory muscles in HGA individuals (vertical growth pattern) was smaller than that in NGA and LGA individuals [17,37, 38, 39]. Moreover, the muscle morphology in HGA individuals results in relatively lower masticatory force or function, which leads to a reduction in cortical bone thickness at the angle of mandible. Thus, mandibular cortical bone width in HGA patients is thinner than that in LGA patients; this is also true for the alveolar bone thickness [36,40, 41, 42]. Thus, it may be stated that the high frequency of HGA patients with MAF is due to the different morphology of their muscles of mastication (smaller cross-sectional area) and thinner cortical bone at the angle of mandible compared with other individuals.

In this study, the mean ramus height was significantly shorter in MAF patients, indicating that short ramus height increases the risk of MAF. Each 1 mm increase in ramus height decreased the odds of MAF (compared with non-MAF) by 42%. Since the linear measurements made on reconstructed CT images are not highly accurate [32], the 42% value cannot be definitely relied on. Similar studies regarding the effect of this parameter on MAF are scarce. Elias *et al.* [7] found significantly shorter ramus height in MAF patients, which was in accordance with the present findings. Slight differences in reported values may be due to different measurement methods. For instance, Elias *et al.* [7] measured the ramus height from the condylar neck width to the gonion. Also, different CT scanners, zooming, and different races and ethnic groups may be responsible for variations of the results.

Elias *et al.* [7] reported significantly higher mean condylar neck width in the MAF group. They justified that greater width results in transfer of *locus minoris resistentiae* to the mandibular angle site and subsequent MAF. However, the two groups were not significantly different regarding this parameter in the present study.

The minimum ramus width and ramus length were also measured in the present study, which have not been previously evaluated in any study. The results showed that the minimum ramus width was significantly smaller in the MAF group, which is a novel finding not reported in any previous study. This finding may indicate that risk of MAF may be lower in subjects with higher minimum ramus width. In explaining this finding, it may be stated that reduction in width may be accompanied by reduction in thickness (particularly in the condyle and cortical bone), which may subsequently increase the risk of fracture. However, further studies are required to assess the cortical bone thickness in patients with lower minimum ramus width. Also, the correlation of minimum ramus width with other fractures in this region, such as condylar and condylar neck fractures should be assessed. Furthermore, by a reduction in width, the applied load is distributed in a smaller surface, and may increase the risk of fracture. Reduction in ramus width may increase stress accumulation at the angle, and increase the risk of mandibular angle fractures. However, all these hypotheses should be tested in future studies.

Keen [43] reported that the GA size increased by an increase in mandibular length. However, the difference in mandibular length was not significant between the two groups in the present study. This controversy in the results may be due to differences in sample size since Keen [43] evaluated 262 patients, versus 118 patients in the present study.

It has been reported that individuals with hyperdivergent facial pattern have a shorter ramus height and larger GA [44, 45, 46]. Also, Mangla *et al.* [45] reported significantly smaller ramus width in hyperdivergent individuals. Thus, it may be assumed that hyperdivergent individuals may be more susceptible to MAF. However, other factors may also be involved in this respect, which call for further investigations on this relationship. Given that this relationship is confirmed, it can be of great help in the clinical setting. The surgeon can detect long-face individuals from their facial properties. A long-face morphology is often characterized by several classic characteristics such as increased height of the facial third, large GA, depressed nasolabial fold, excessive tooth show and maxillary gingival show, incompetent lips, narrow palate, and posterior cross-bite and anterior open-bite in some cases [47]. Thus, in case of encountering patients with mandibular trauma, the surgeon should suspect MAF by noticing an increased GA, short ramus height, and small minimum ramus width (which are the characteristics of hyperdivergent individuals).

Third molar impaction attenuates the mandible due to decreased bone volume since it occupies a space in bone. However, risk of MAF in patients with erupted third molars has not been confirmed [5,48, 49, 50]. In the present study, the frequency of impacted third molars at the fracture site was significantly higher in the MAF group. The results also revealed that presence of impacted third molar increased the risk of MAF (compared with non-MAF) by 5.5 times. However, the frequency of erupted third molars was higher in the non-MAF group. Thus, MAF was not significantly correlated with the presence of erupted third molars. Iida *et al.* [48] reported significantly higher prevalence of MAF in patients with impacted third molars, but found no significant correlation between the presence of erupted third molars and prevalence of MAF, which was similar to the present findings. Giovacchini *et al.* [20] found that presence of third molars, irrespective of their eruption status, increased the relative risk of MAF by 1.90 times (95% CI: [1.47, 2.46]). In contrast to the present study, they evaluated erupted and impacted third molars in one group. According to the present results, it is recommended to surgically extract the impacted third molars in patients susceptible to MAF to decrease the odds of this type of fracture in them.

Further multi-center studies with a larger sample size and equal ratio of men and women are required to assess the correlation of other anatomical and radiographic indices with MAF. More and new radiographic indices should be evaluated in future studies. Also, radiomorphometric indices of hyperdivergent patients with MAF should be further investigated.

## 5 Conclusion

This study showed that patients with large GA, short ramus height, small minimum ramus width, and impacted third molar were more susceptible to MAF. Extraction of impacted third molars and further protection in those who practice contact sports are recommended. Also, surgeons can use these indices to predict the risk of MAF in trauma patients with such facial characteristics, and make a diagnosis by simpler radiographic modalities such as panoramic radiography. This can help faster management of such fractures and prevention of complications.

## Declarations

### Author contribution statement

Aida Karagah; Maryam Tofangchiha: Conceived and designed the experiments; Wrote the paper.

Reza Tabrizi; Romeo Patini: Contributed reagents, materials, analysis tools or data.

Fatemeh Pourahmadali: Performed the experiments

Ahad Alizadeh: Analyzed and interpreted the data.

### Funding statement

This research did not receive any specific grant from funding agencies in the public, commercial, or not-for-profit sectors.

### Data availability statement

Data included in article/supp. material/referenced in article.

### Declaration of interest’s statement

The authors declare no conflict of interest.

### Additional information

No additional information is available for this paper.

## References

[1] Shilo D., Elias Y.B., Capucha T., Blanc O., Emodi O., Rachmiel A. (2019). Craniofacial morphometric features associated with pericondylar fractures of the mandible. J. Craniofac. Surg..

[2] Shroff N., Motghare P., Kumbhare S., Kalaskar A. (2020). Correlation of mandibular gonial angle and mandibular angle fracture: a radiographic study. J. Indian Acad. Oral Med. Radiol..

[3] Farzan R., Farzan A., Farzan A., Karimpour M., Tolouie M. (2021). A 6-year epidemiological study of mandibular fractures in traumatic patients in North of Iran: review of 463 patients. World J. Plast. Surg..

[4] Radabaugh J.P., Horn A.V., Chan S.A., Shelton J.M., Gal T.J. (2017). Patient compliance following isolated mandibular fracture repair. Laryngoscope.

[5] Dhara V., Kamath A.T., Vineetha R. (2019). The influence of the mandibular gonial angle on the occurrence of mandibular angle fracture. Dent. Traumatol..

[6] Al-Moraissi E.À., El-Sharkawy T.M., El-Ghareeb T.I., Chrcanovic B.R. (2014). Three-dimensional versus standard miniplate fixation in the management of mandibular angle fractures: a systematic review and meta-analysis. Int. J. Oral Maxillofac. Surg..

[7] Bereznyak Elias Y., Shilo D., Emodi O., Noy D., Rachmiel A. (2018). The relation between morphometric features and susceptibility to mandibular angle fractures. J. Craniofac. Surg..

[8] Tiwari P., Bera R.N., Chauhan N. (2020). Magnitude of gonial angle influence on the commonness of mandibular angle fractures. Ann Maxillofac Surg.

[9] Semel G., Emodi O., Ohayon C., Ginini J.G., Rachmiel A. (2020). The influence of mandibular gonial angle on fracture site. J. Oral Maxillofac. Surg..

[10] Yoon W.J., Kim S.G., Oh J.S., You J.S., Lim K.S., Shin S.M., Kim C.M. (2014). A clinical study of mandibular angle fracture. Maxillofac Plast Reconstr Surg.

[11] Hasegawa T., Sadakane H., Kobayashi M., Tachibana A., Oko T., Ishida Y. (2016 Sep 1). A multi-center retrospective study of mandibular fractures: do occlusal support and the mandibular third molar affect mandibular angle and condylar fractures?. Int. J. Oral Maxillofac. Surg..

[12] Meisami T., Sojat A., Sàndor G.K., Lawrence H.P., Clokie C.M. (2002). Impacted third molars and risk of angle fracture. Int. J. Oral Maxillofac. Surg..

[13] Brucoli M., Boffano P., Pezzana A., Benech A., Corre P., Bertin H. (2019). The "European mandibular angle" research project: the epidemiologic results from a multicenter European collaboration. J. Oral Maxillofac. Surg..

[14] Koshy S., Rajan R., Bonanthaya K., Prathap A., Wilson A.A. (2021). Relationship between manbibular angle fractures and mandibular third molars-An observational study. Ann. Oral Maxillofac Surg..

[15] Panneerselvam E., Prasad P.J., Balasubramaniam S., Somasundaram S., Raja K.V., Srinivasan D. (2017). The influence of the mandibular gonial angle on the incidence of mandibular angle fracture-A radiomorphometric study. J. Oral Maxillofac. Surg..

[16] Fuselier J.C., Ellis E.E., Dodson T.B. (2002). Do mandibular third molars alter the risk of angle fracture?. J. Oral Maxillofac. Surg..

[17] Sirin Y., Yildirimturk S., Ay N., Gencel B. (2020). The influence of the gonial angle on the initial biomechanical stability of the plate osteosynthesis in polyurethane mandibles with angle fractures. J. Craniofac. Surg..

[18] Al-Moraissi E.A., Ellis E. (2014). What method for management of unilateral mandibular angle fractures has the lowest rate of postoperative complications? A systematic review and meta-analysis. J. Oral Maxillofac. Surg..

[19] Patel N., Kim B., Zaid W. (2016). A detailed analysis of mandibular angle fractures: epidemiology, patterns, treatments, and outcomes. J. Oral Maxillofac. Surg..

[20] Giovacchini F., Paradiso D., Bensi C., Belli S., Lomurno G., Tullio A. (2018). Association between third molar and mandibular angle fracture: a systematic review and meta-analysis. J. Cranio-Maxillo-Fac. Surg..

[21] Ruela W.S., de Almeida V.L., Lima-Rivera L.M., Santos P.L., Porporatti A.L., de Freitas P.H.L. (2018). Does an association exist between the presence of lower third molar and mandibular angle fractures?: a meta-analysis. J. Oral Maxillofac. Surg..

[22] Tevepaugh D.B., Dodson T.B. (1995). Are mandibular third molars a risk factor for angle fractures? A retrospective cohort study. J. Oral Maxillofac. Surg..

[23] Bhullar M.K., Uppal A.S., Kochhar G.K., Chachra S., Kochhar A.S. (2014). Comparison of gonial angle determination from cephalograms and orthopantomogram. Indian J. Dent..

[24] Leversha J., McKeough G., Myrteza A., Skjellrup-Wakefiled H., Welsh J., Sholapurkar A. (2016). Age and gender correlation of gonial angle, ramus height and bigonial width in dentate subjects in a dental school in Far North Queensland. J Clin Exp Dent.

[25] Rai A. (2021).

[26] Upadhyay R.B., Upadhyay J., Agrawal P., Rao N.N. (2012). Analysis of gonial angle in relation to age, gender, and dentition status by radiological and anthropometric methods. J. Forensic Dent. Sci..

[27] Han L., Long T., Tang W., Liu L., Jing W., Tian W. (2017). Correlation between condylar fracture pattern after parasymphyseal impact and condyle morphological features: a retrospective analysis of 107 Chinese patients. Chin Med J (Engl)..

[28] Toman H.A., Nasir A., Hassan R., Hassan R. (2011). Skeletal, dentoalveolar, and soft tissue cephalometric measurements of Malay transfusion-dependent thalassaemia patients. Eur. J. Orthod..

[29] Tripathi T., Gupta P., Rai P., Sharma J., Gupta V.K., Singh N., Verma M. (2019). Longitudinal evaluation of the association between Insulin-like growth factor-1, Bone specific alkaline phosphatase and changes in mandibular length. Sci. Rep..

[30] Haas D.W., Martinez D.F., Eckert G.J., Diers N.R. (2001). Measurements of mandibular length: a comparison of articulare vs condylion. Angle Orthod..

[31] Zhou H.H., Liu Q., Cheng G., Li Z.B. (2013). Aetiology, pattern and treatment of mandibular condylar fractures in 549 patients: a 22-year retrospective study. J. Cranio-Maxillo-Fac. Surg..

[32] Markose E., Vikraman B., Veerabahu M. (2009). Three dimensional CT reconstruction: a comparison between 2D, 3D CT and original anatomical structures. J Maxillofac Oral Surg.

[33] Afrooz P.N., Bykowski M.R., James I.B., Daniali L.N., Clavijo-Alvarez J.A. (2015). The epidemiology of mandibular fractures in the United States, Part 1: a review of 13,142 cases from the US national trauma data bank. J. Oral Maxillofac. Surg..

[34] Amarista Rojas F.J., Bordoy Soto M.A., Cachazo M., Dopazo J.R., Vélez H. (2017). The epidemiology of mandibular fractures in Caracas, Venezuela: incidence and its combination patterns. Dent. Traumatol..

[35] Xiao D., Gao H., Ren Y. (2011). Craniofacial morphological characteristics of Chinese adults with normal occlusion and different skeletal divergence. Eur. J. Orthod..

[36] Osato S., Kuroyama I., Nakajima S., Ogawa T., Misaki K. (2012). Differences in 5 anatomic parameters of mandibular body morphology by gonial angle size in dentulous Japanese subjects. Ann. Anat..

[37] Ariji Y., Kawamata A., Yoshida K., Sakuma S., Nawa H., Fujishita M. (2000). Three-dimensional morphology of the masseter muscle in patients with mandibular prognathism. Dentomaxillofacial Radiol..

[38] Kiliaridis S., Kälebo P. (1991). Masseter muscle thickness measured by ultrasonography and its relation to facial morphology. J. Dent. Res..

[39] van Spronsen P.H., Weijs W.A., Valk J., Prahl-Andersen B., van Ginkel F.C. (1992). A comparison of jaw muscle cross-sections of long-face and normal adults. J. Dent. Res..

[40] Ono A., Motoyoshi M., Shimizu N. (2008). Cortical bone thickness in the buccal posterior region for orthodontic mini-implants. Int. J. Oral Maxillofac. Surg..

[41] Sharma N.S., Shrivastav S.S., Hazarey P.V. (2012). Relationship among types of growth patterns, buccolingual molar inclination and cortical bone thickness of the mandible: a CT scan study. J. Indian Orthod. Soc..

[42] García-Morales P., Buschang P.H., Throckmorton G.S., English J.D. (2003). Maximum bite force, muscle efficiency and mechanical advantage in children with vertical growth patterns. Eur. J. Orthod..

[43] Keen J.A. (1945). A study of the angle of the mandible. J. Dent. Res..

[44] Lemes C.R., Tozzi C.F., Gribel S., Gribel B.F., Venezian G.C., do Carmo Menezes C. (2021). Mandibular ramus height and condyle distance asymmetries in individuals with different facial growth patterns: a cone-beam computed tomography study. Surg. Radiol. Anat..

[45] Mangla R., Singh N., Dua V., Padmanabhan P., Khanna M. (2011). Evaluation of mandibular morphology in different facial types. Contemp. Clin. Dent..

[46] Buschang P.H., Jacob H., Carrillo R. (2013). In Seminars in Orthodontics.

[47] Schendel S.A., Eisenfeld J., Bell W.H., Epker B.N., Mishelevich D.J. (1976). The long face syndrome: vertical maxillary excess. Am. J. Orthod..

[48] Iida S., Hassfeld S., Reuther T., Nomura K., Mühling J. (2005). Relationship between the risk of mandibular angle fractures and the status of incompletely erupted mandibular third molars. J. Cranio-Maxillo-Fac. Surg..

[49] Duan D.H., Zhang Y. (2008). Does the presence of mandibular third molars increase the risk of angle fracture and simultaneously decrease the risk of condylar fracture?. Int. J. Oral Maxillofac. Surg..

[50] Naghipur S., Shah A., Elgazzar R.F. (2014). Does the presence or position of lower third molars alter the risk of mandibular angle or condylar fractures?. J. Oral Maxillofac. Surg..

